# P23-Specific IgY Significantly Reduces Diarrhea and Oocyst Shedding in Calves Experimentally Infected with *Cryptosporidium parvum*

**DOI:** 10.3390/vaccines13020162

**Published:** 2025-02-07

**Authors:** Anabela Mira, Carlos Javier Garro, Paloma de Alba, Demian Monti, Maria Cecilia Lang, Alejandro Vivas, Esteban Medina, Juan Cruz Franco, Álvaro Gutierrez, Leonhard Schnittger, Andrés Wigdorovitz, Viviana Parreño, Marina Bok

**Affiliations:** 1 Bioinnovo S.A., De Los Reseros y Nicolás Repetto S/N, Buenos Aires 1686, Argentina; amira@bioinnovo.com (A.M.); mlang@bioinnovo.com (M.C.L.); avivas@bioinnovo.com (A.V.); emedina@bioinnovo.com (E.M.); jfranco@bioinnovo.com (J.C.F.); 2 Instituto de Patobiología Veterinaria (IP-IPVet), CICVyA, Instituto Nacional de Tecnología Agropecuaria (INTA-CONICET), De Los Reseros y Nicolás Repetto S/N, Buenos Aires 1686, Argentina; garro.carlos@inta.gob.ar (C.J.G.); dealba.paloma@inta.gob.ar (P.d.A.); schnittger.leonhard@inta.gob.ar (L.S.); 3 Consejo Nacional de Investigaciones Científicas y Técnicas (CONICET), Buenos Aires C1425FQB, Argentina; wigdorovitz.andres@inta.gob.ar; 4 INCUINTA, CICVyA, Instituto Nacional de Tecnología Agropecuaria, De Los Reseros y Nicolás Repetto S/N, Buenos Aires 1686, Argentina; monti.demian@inta.gob.ar (D.M.); alvaromatgutierrez@gmail.com (Á.G.)

**Keywords:** *Cryptosporidium parvum*, neonatal diarrhea, calves, IgY, treatment

## Abstract

Background/Objectives: *Cryptosporidium parvum* is a zoonotic enteroparasite causing severe diarrhea in newborn calves, leading to significant economic losses in dairy and beef farming. This study aimed to evaluate whether *C. parvum* p23-specific IgY antibodies could control neonatal calf diarrhea caused by *C. parvum*. Methods: A recombinant immunogen comprising the p23 protein fused to the antigen-presenting cell homing (APCH) molecule was expressed using the baculovirus system. Hens were immunized with the APCH-p23 immunogen, and the resulting IgY was spray-dried for treatment use. Eight newborn calves were included in the study and received commercial colostrum within the first 12 h of life. Four calves were treated with 20 g of powdered egg containing IgY (p23-specific IgY titer of 256 in milk) twice daily for 7 days. The remaining four calves received regular non-supplemented milk. All calves were orally infected with 6 million oocysts and monitored for 21 days. Results: Calves treated with p23-specific IgY exhibited significantly reduced diarrhea duration (3.5 vs. 7.5 days; *p* = 0.0397) and oocyst shedding duration (6.50 vs. 12 days; *p* = 0.0089). In addition, the total number of excreted oocysts, as measured by the change of the area under the curve (AUC), was significantly reduced in the treated group (14.25 vs. 33.45; *p* = 0.0117). Although the onset of diarrhea was delayed (3.5 to 6.5 days post-infection; *p* = 0.1840), and diarrhea severity was reduced (24.25 to 17 AUC; *p* = 0.1236), both parameters were not statistically significant. Conclusions: P23-specific IgY antibodies effectively reduced the *C. parvum*-induced duration of diarrhea in experimentally infected calves. These findings highlight the potential of this passive treatment as a promising strategy for controlling neonatal calf diarrhea.

## 1. Introduction

*Cryptosporidium* species are unicellular eukaryotic parasites of the phylum Apicomplexa [1]. Of these, *Cryptosporidium parvum* is considered the principal pathogen associated with neonatal calf diarrhea (NCD) in cattle worldwide [2]. As a zoonotic species, *C. parvum* is particularly relevant from a One Health perspective because it also causes cryptosporidiosis in immunocompetent patients and the elderly and high mortality in toddlers in some regions of the world [3,4]. The prevalence of *C. parvum* in dairy calves is highly variable, ranging from 0% to more than 40% [5]. In Argentina, the prevalence of calves up to 20 days of age varied between 25.2% and 42.5% depending on the region, as shown in at least five independent large-scale epidemiological studies reviewed in de Alba et al., 2023 [6,7,8].

*Cryptosporidium* parasites display a monoxenous life cycle, including asexual and sexual reproduction. Infected calves shed large numbers of oocysts in their feces, contaminating the environment. Typically, oocysts remain infective for several months in water and moist environments and are resistant to high concentrations of chlorine [9]. Transmission to a new host occurs via the fecal–oral route, either by direct contact with contaminated feces or indirectly by ingestion of water or food contaminated with oocysts. Once ingested by the host, oocysts excyst and invade the cells of the gastrointestinal tract. Successive cycles of asexual and sexual reproduction generate massive numbers of oocysts that are excreted into the environment with feces [10,11]. Among surface antigens that have been described to be expressed in the different parasite stages, there is p23, a 23 kDa glycoprotein, which is located on the surface of oocyst but also on the surface of sporozoites and merozoites [12]. P23 is antigenically conserved across geographically diverse isolates, has neutralization-sensitive epitopes, and is deposited in trails during the initial stages of infection [13,14]. As a vaccine, p23 has been used in pregnant heifers to produce hyperimmune colostrum to protect calves against cryptosporidiosis [15]. In addition, p23 has been used as an immunogen to generate p23-specific IgY for the passive immunization of animals and humans against cryptosporidiosis with promising results [12,16,17].

A novel approach for effective bovine immunization is fusing the vaccine candidate p23 to the antigen-presenting cell homing (APCH) molecule. The APCH molecule is a single-chain antibody against an invariant of the major histocompatibility complex class II (MHC-II) epitope, which targets the vaccine candidate to the antigen-presenting cell (APC) facilitating the development of a specific adaptive immune response. The development and testing of APCH was originally conducted in swine; however, subsequent studies demonstrated its capacity for cross-reactivity with MHC-II in other species, including bovines [18]. The baculovirus–insect cell expression system was chosen to express the APCH-p23 immunogen as it is one of the most widely used tools for the high-level expression of heterologous proteins in eukaryotic hosts. Besides the advantage of high-level expression, the insect cells are capable of post-translational modifications similar to those found in mammalian cells. Furthermore, the baculovirus system is characterized by its safety, ease of use, and its capacity for straightforward scaling up [19].

Currently, no efficient treatments are available for the control of bovine cryptosporidiosis. Treatment with the coccidiostat halofuginone has important limitations, such as its toxicity with slightly increased doses, while the antibiotic paromomycin is not available in Argentina. Recently, a subunit vaccine based on recombinant gp40 of *C. parvum* for use in pregnant cows has been approved but is unavailable in many South American countries [20]. Furthermore, passive immunization using egg yolk, sometimes referred to as IgY technology, has been studied for many viral and bacterial pathogens [21,22,23]. IgY technology is based on the inoculation of hens with the immunogen of interest to produce antigen-specific IgY immunoglobulins. The generated antigen-specific IgY is used to formulate a product that can be used to prevent or treat the corresponding infection of a pathogen. The IgY technology has already been successfully developed against several diarrhea-causing pathogens, such as rotavirus and coronavirus [21,22], which has resulted in a commercial product (IgY DNT, Bioinnovo S.A.) that has been registered and is available in Argentina and several other countries [24].

This study aimed to evaluate the efficacy of a *C. parvum* p23-specific IgY-based product (p23-IgY) to control diarrhea and oocyst shedding in calves experimentally infected with *C. parvum*.

## 2. Materials and Methods

### 2.1. Cells and Recombinant Antigens

*Cryptosporidium* p23 protein was fused to the APCH molecule and recombinantly expressed for subsequent use as an immunogen in hens. APCH-p23 antigen, including a 6xHis tag, was produced in the baculovirus–insect cells system (BAC to BAC technology). Briefly, the APCH-p23 sequence was cloned into the pFastBac1 (Thermo Fisher, USA) vector. Competent DH10Bac *Escherichia coli*, containing the complete genome of baculovirus AcMNPV, were transformed and transposed to obtain the recombinant bacmid. Sf9 (*Spodoptera frigiperda*) insect cells were transfected with the bacmid DNA to obtain a recombinant baculovirus that includes the sequence for expression of the APCH-p23 antigen. Serial passages in Sf9 cells of the recombinant baculovirus were conducted to amplify the viral stock until a minimum titer of 10^7^ plaque-forming units (PFUs)/mL. Viral production was completed by infecting Sf9 cells (1.1 × 10^6^ cells/mL) with the previous viral passage using a multiplicity of infection (MOI) of 0.05 in Sf-900 medium supplemented with 10% FBS for 3 days at 28 °C with 150 rpm shaking.

APCH-p23 proteins were produced by infection of SF9 cells (1.5–2 × 10^6^ cells/mL) with the viral stock at a MOI of 5 using the same conditions as described before. The expressed fusion protein is secreted to the infection supernatant after 5–7 days, and APCH-p23 protein is collected after a centrifugation step (5000× *g*, 15’ and 4 °C) and stored at 4 °C until immunogen formulation.

The p23 antigen was produced in the *E. coli* system for ELISA assays. The p23 gene was directionally subcloned from the pFastBac vector into the pRSetA plasmid utilizing BamHI and HindIII restriction enzymes. Following the transformation of the *Escherichia coli* DH5α strain, confirmation of the correct assembly of the pRSetA-p23 construct was achieved by colony PCR analysis. Subsequently, sequencing was conducted to validate the integrity of the construct. For expression of the p23 antigen, *E. coli* BL21 cells were transformed with recombinant plasmid, and a transformed colony was grown in LB medium and induced at OD 0.6–0.8 with IPTG (final concentration 1 mM). Culture was incubated at 28 °C in a shaker at 200 rpm overnight (ON). Bacterial culture was centrifuged at 5000× *g* for 10 min, and supernatant was discarded. The pellet was resuspended in a lysis buffer (Tris 50 mM pH8, NaCl 100 mM, Triton X100 0.05%, and PMSF 1 mM), sonicated, and centrifuged 30,000× *g* for 30 min at 4 °C. The expressed protein was purified from the supernatant by IMAC (immobilized metal affinity chromatography) using Ni-NTA affinity resin (Amintra, Abcam, Cambridge, United Kingdom) following the manufacturer’s instructions.

### 2.2. Quality Control of APCH-p23 Antigen

The production of APCH-p23 protein was evaluated by Western blot analysis. Briefly, 15 µL of the sample (supernatant + loading buffer) was run in a 12% polyacrylamide gel to separate the proteins and transferred to a PVDF membrane. The membrane was blocked with 3% milk solution in TBS-Tween 0.1%, incubated with an anti-His-tag antibody (Ab) produced in mice (GenScript, New Jersey, USA) at a 1/2000 dilution and an HRP-tagged anti-mouse Ab (Jackson Immuno Research, Pennsylvania, USA) at a 1/5000 dilution. At each reaction step, the membrane was incubated at 37 °C for 1 h. APCH-p23 (~ 45 kDa) was detected using ECL (Super Signal West Pico PLUS Chemiluminescent Substrate, Thermo Scientific, Massachusetts, USA) and visualized under UV light.

The quantity of the recombinant protein in the supernatant was estimated by Dot Blot. Serial dilutions of supernatant were transferred to a nitrocellulose membrane using a vacuum pump. Then, the blocking and Ab incubation steps were performed in the same manner as described before for the Western blot. APCH-E2 (E2, Bovine Viral Diarrhea Virus protein) was included as a positive calibration standard for each assay for relative quantification of the protein of interest. The protein used for standard curves was quantified by a statistically validated ELISA [25]. Quantity estimation was conducted by comparing the OD values of the dilutions of the target protein with those of the standard curve.

### 2.3. Cryptosporidium Parvum P23-Specific IgY

An experimental vaccine was formulated using the unpurified expression supernatant containing the APCH-p23 with Montanide 51 VG adjuvant (Seppic, Courbevoie, France) in equal proportions (1:1 ratio). Prior to vaccine formulation, the supernatant was subjected to tangential flow filtration (TFF), filtered through a 0.2 µm membrane, and underwent sterility testing. The emulsification of the oil and aqueous phases was carried out using a T25 digital Ultraturrax (IKA, Staufen, Germany) immediately prior to each immunization. Fifty laying hens of the white Leghorn breed were immunized intramuscularly with 0.5 mL doses containing approximately 3 µg of recombinant APCH-p23. The immunization schedule included doses on days 0, 21, 45, and 70, followed by additional booster doses every 60 days to sustain IgY antibody levels in the serum and egg yolk. Antibody response to the immunogen was evaluated by measuring p23-specific IgY titers in the serum of five randomly chosen hens using a p23-ELISA (see Section 2.4).

To produce egg powder batches, eggs collected from the immunized hens were processed using an egg-breaking machine (Pelbo, Brugherio, Italy) and then spray-dried (FlexPump, Galaxie S.A., Buenos Aires, Argentina). The IgY antibody content in the resulting powder was assessed by ELISA. The batches were stored at a controlled temperature of 18–24 °C until further use. Microbiological quality assurance was performed in accordance with local regulations for animal feed, confirming the absence of *Salmonella* in 25 g, total mesophilic aerobes below 1 × 10⁶ CFU/g, total coliforms below 1 × 10³ CFU/g, and no detection *of E. coli* in 1 g [22,24].

### 2.4. P23 Avian IgY Specific Antibody ELISA

IgY Ab titers against recombinant p23 expressed in *E. coli* were measured in egg powder, chicken sera, supplemented milk, and calf feces using an indirect ELISA. In this assay, 96-well high-binding ELISA plates (ES08, IVEMA Desarrollos, Buenos Aires, Argentina) were coated with 500 ng of recombinant p23 per well, diluted in a carbonate-bicarbonate buffer (pH 9.6). After blocking with 10% non-fat milk, serial four-fold sample dilutions were added and incubated at 37 °C for 1 h. Following incubation, plates were washed four times with PBS containing 0.05% Tween-20 and then incubated for 1 h with a peroxidase-conjugated polyclonal goat anti-chicken IgY antibody (Abcam, Cambridge, United Kingdom) diluted 1:5000 in 0.05% PBS-Tween20. ABTS was used for ELISA development. The antibody titer of each sample was calculated as the reciprocal of the highest dilution that produced an OD value above the assay’s cut-off. The cut-off was defined as the mean OD of blank wells plus or minus 3 standard deviations (SD). Samples that tested negative at a 1:4 dilution were assigned a titer of 2 to calculate the geometric mean titers (GMTs).

### 2.5. Efficacy Study in Calves Experimentally Challenged with C. parvum

#### 2.5.1. Production of *C. parvum* Inoculum

Fecal samples were collected from naturally infected calves from a dairy farm located in Buenos Aires, Argentina. Oocyst-positive fecal samples were detected by lateral flow immuno-chromatography. Subsequently, oocysts were purified from fecal samples as described [26]. Briefly, two serial sucrose gradients were followed by one cesium chloride gradient to obtain purified oocysts, which were diluted in PBS and stored at 4 °C until use. Oocysts were counted using a Neubauer chamber ensuring a CV (coefficient of variation) of under 15%. *Cryptosporidium* species was determined by PCR-RFLP (PCR-restriction fragment length polymorphism). Briefly, the amplification of the *Cryptosporidium* 18S rRNA gene by PCR was followed by digestion using *Ssp*I, *Vsp*I, and *Mbo*II restriction enzymes [6,27]. The specific band pattern allows us to determine the *Cryptosporidium* species (*C. parvum*: Ssp I: 649, 254, and 108/119 bp; VspI: 625/628, 102/104, and 28 bp; MboII: 771 and 63 bp).

A total of 50 million purified oocysts were utilized as the inoculum to infect a newborn calf, thereby generating a new batch of fresh purified oocysts that were subsequently employed to infect calves in the efficacy study. Oocysts from this calf were purified and counted as described above. Before inoculation, the viability of the oocysts was tested using an excystation assay using taurocholic acid as described [28].

#### 2.5.2. Experimental Design to Test the Efficacy of the Milk Supplemented with P23 Egg Yolk Powder to Prevent *C. parvum* Infection and Diarrhea

**Figure 1:**
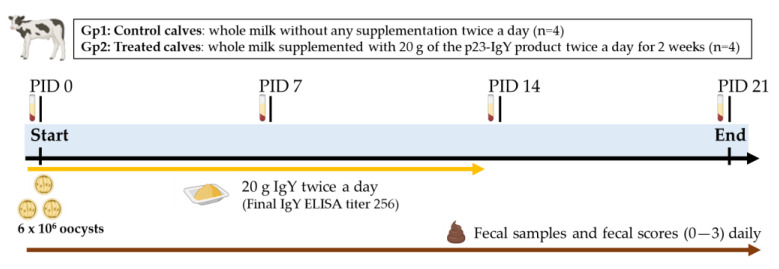
Experimental design of the efficacy study. Gp = group; PID = post-inoculation day.

The experimental design to evaluate the effectiveness of milk supplemented with p23-enriched egg yolk powder in preventing *Cryptosporidium parvum* infection and diarrhea is illustrated in Figure 1. Eight newborn male Holstein calves from the same dairy farm were used. The calves were separated from their dams immediately after birth, prior to nursing, and transported to the isolation facility within the first 4 h of life. They were housed individually in isolation pens under a strict management protocol. To minimize the risk of metabolic disorders and bacterial infections, all calves received vitamins, selenium, and prophylactic antibiotic treatment. Within the first 12 h of life, each calf was fed 2 L of Colostrum 100™ (SCCL, Saskatoon, Canada) as a substitute for natural colostrum.

For routine feeding, the calves were provided 2 L of antibody-free milk twice daily, supplemented with Ruter VG (ACA, San Nicolás, Argentina) as solid feed. The milk used in the study was a commercially sterilized bovine milk containing 3% fat, intended for human consumption (Verónica, Buenos Aires, Argentina). The calves were randomly allocated into two feeding groups: Group 1 (Gp1; n = 4), which received unsupplemented milk, and Group 2 (Gp2; n = 4), which was fed milk supplemented twice daily with 20 g of p23-IgY powder, achieving a final ELISA IgY antibody titer of 256 in the milk.

At 48–72 h after birth (designated as post-inoculation day, PID 0), all calves were orally inoculated with 6 × 10⁶ viable *C. parvum* oocysts, a dose previously shown to induce diarrhea and oocyst shedding in 100% of control calves. Calves in the IgY treatment group (Group 2) began receiving supplemented milk 24 h before inoculation (PID -1). After PID 15, all calves in both groups were switched to unsupplemented milk (end of treatment).

Daily monitoring included clinical signs of diarrhea, oocyst shedding, and elevated rectal temperature (defined as >38.5 °C). Fecal consistency was scored following the calf scoring system of the University of Madison-Wisconsin, where 0 = solid, 1 = paste, 2 = watery, and 3 = severe watery. A score of 2 or higher indicated diarrhea. The scoring was conducted independently and blinded by two trained technicians. Fecal samples were collected daily before and after inoculation to quantify oocyst shedding by qPCR. Serum samples were taken on the day of inoculation and weekly thereafter (PID 7, 14, and 21). Serum and fecal levels of IgM, IgA, and IgG antibodies against p23 were measured using isotype-specific ELISA.

#### 2.5.3. *Cryptosporidium parvum* Detection by Quantitative Real-Time PCR

*Cryptosporidium* oocyst shedding was measured by a qPCR targeting the 18S rRNA gene of *C. parvum,* as previously described [29]. Briefly, fecal samples were diluted 1:10 in PBS, and total DNA was extracted using a PuriPrep-SUELO kit (Inbio Highway, Tandil, Argentina) following the manufacturer’s instructions. The qPCR assay was performed in a BioRad CFX 96 Thermocycler using BioRad SsoAdvanced Universal Probes Supermix. The standard curve was optimized using known concentrations of plasmids containing the cloned 18S rRNA target sequence. Primers, probe, and real-time PCR conditions were used as described elsewhere [29].

#### 2.5.4. Bovine Isotype-Specific P-23 Ab ELISAs

The titers of IgM, IgA, and IgG Ab to the *Cryptosporidium* p23 antigen were quantified in calf sera, feces, and commercial colostrum. Specific Abs were detected by an indirect ELISA, which was especially standardized for this study. For the assay, 96-well high-binding ELISA plates (ES08, IVEMA Desarrollos, Buenos Aires, Argentina) were coated overnight at 4 °C with 500 ng of purified recombinant p23 protein, expressed in *E. coli*, diluted in carbonate–bicarbonate buffer (pH 9.6). After coating, plates were blocked with 10% non-fat milk (La Serenísima, Mastellone Hnos, Buenos Aires, Argentina). Serial four-fold dilutions of the samples were then added and incubated at 37 °C for 1 h. The ELISA plate was washed four times and incubated with a peroxidase-labeled polyclonal goat anti-bovine IgM, IgA, or IgG (Bethyl Laboratories INC., Montgomery, TX, USA) in a 1/3000 dilution at 37 °C for 1 h. ABTS were used for ELISA development. This ELISA assay was statistically validated, and a cut-off value of 0.125 was defined, which corresponded to a test sensitivity of 100% and a specificity of 97.62%. Antibody titers for each sample were determined as the reciprocal of the highest dilution that produced an OD value above the established cut-off.

#### 2.5.5. Differential Diagnosis

Rotavirus and coronavirus diagnosis were performed to discard the presence of infectious agents causative for diarrhea. The presence of both rotavirus and coronavirus antigens in fecal samples was evaluated by lab-made ELISA [22,30].

### 2.6. Statistical Analysis

The efficacy study’s statistical analysis was performed as detailed below.

Calves were born from eight different mothers and enrolled randomly in each experimental group as independent units. The average amount of shed oocysts was estimated based on the 18S rRNA gene copy number determined each day from PID 1 to PID 14 for the control and treatment groups, respectively. This allowed us to estimate the average of the total number of oocysts shed from PID 1 to PID 14 for each study group, which was expressed as the area under the curve (AUC). The AUC for diarrhea scores and oocyst shedding was determined using the spline method through the AUC function of the DescTools package in RStudio.

The mean day of onset, the mean duration of oocyst shedding and diarrhea, and the average total of shed oocysts and diarrhea from PID 1 to 14 were compared using the Student’s t-test with Welch’s correction when the normality of residues was met, but the assumption of homoscedasticity was not met. The Wilcoxon rank sum test was used when the normality and homoscedasticity assumptions were not met.

For IgY titers in hens’ serum and bovine isotype-specific antibody titers in calf serum and feces, the data were log10-transformed. Serum samples negative at a dilution of 1:4 were assigned an arbitrary antibody titer of 2 (log10 = 0.3) for the calculation of geometric mean titers (GMTs) and arithmetic means. IgM, IgA, and IgG titers in serum (PID 0 to PID 21), along with oocyst shedding (quantified as 18S rRNA gene copy numbers by qPCR) and diarrhea scores (PID 1 to PID 21), were analyzed using a two-way repeated-measures ANOVA over time. Statistical significance was considered at *p* < 0.05.

All statistical analyses were performed using Infostat software (Version 29-09-2020) linked to R or RStudio (Version 1.4.1717, RStudio, PBC). Additionally, the statistical power for all evaluated parameters was calculated with G-Power software (Version 3.1.9.7).

## 3. Results

### 3.1. Production of C. parvum P-23 Recombinant Antigen

**Figure 2:**
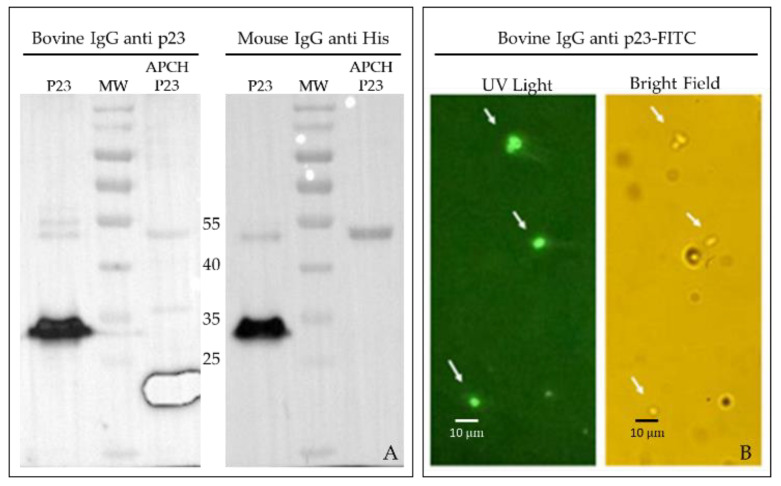
Western blot analysis of p23 antigens. (**A**) p23 of ~30 kDa expressed in *E. coli* and APCH-p23 of 44.6 kDa expressed in the baculovirus system reacted with bovine immune serum derived from cows immunized with APCH-p23 antigen (**left panel**) or commercially obtained mouse anti-his-tag IgG (**right panel**); (**B**) Purified oocysts reacted with FITC-labeled purified-bovine immune serum; view under fluorescence microscope using UV light (**left panel**) or bright field (**right panel**); arrows indicate the oocysts; 400×.

The APCH-p23 was secreted into the culture supernatant, achieving an expression level of 10 mg/L. SDS-PAGE gel electrophoresis confirmed that the apparent corresponded with the calculated molecular weight of 44.6 kDa. To study the immune response against *C. parvum*, the p23 antigen lacking the APCH moiety was expressed for use as an antigen in isotype-specific ELISA assays. A yield of 3.75 mg of p23 was obtained in the supernatant per liter of culture. Although some p23 antigen remained in the pellet, subsequent purification steps were carried out using only the supernatant. The p23 antigen could be confirmed as electrophoretic mobility corresponding with the expected molecular weight of ~23 kDa (Figure 2). P23-specific bovine serum was generated and used to confirm the identity of p23 by Western blot (Figure 2A) and, after labeling with FITC, to demonstrate the presence of p23 on *C. parvum* oocysts (Figure 2B). In addition, the specificity of IgY from immunized hens for *C. parvum* p23 on the oocyst surface was confirmed by ELISA using oocysts from fecal samples (Supplementary Materials Part S2).

### 3.2. Chicken Egg Powder Enriched in Anti-C. parvum P-23 IgY

**Figure 3:**
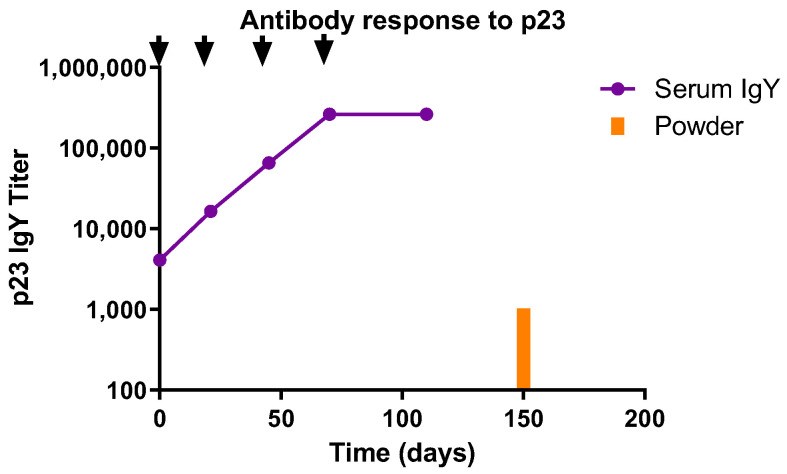
IgY Ab response in sera of laying hens immunized with *C. parvum* APCH-p23 antigen at different times and the final p23-IgY titer in the powder batch as determined by p23-specific ELISA. Arrows indicate immunization time points.

Fifty laying hens were immunized four times with the APCH-p23 immunogen as described in the M&M section. The immunization procedure resulted in a final p23-specific IgY ELISA titer of 262,144 (GMT) in hen sera. A total of 15 kg of egg powder was obtained with a p23-specific IgY ELISA titer of 1024 (as determined in a suspension of 10 mg/mL of powder in PBS) (Figure 3). Eggs from immunized hens were collected daily and stored at a controlled temperature (20–24 °C). At two-week intervals, whole eggs were sanitized, crushed, and spray-dried at the Bioinnovo manufacturing facility. The egg powder batch passed all microbiological controls for animal consumption. IgY powder was stored at a controlled room temperature (22–24 °C) until use.

### 3.3. Generation of C. parvum Oocyst Inoculum and Species Verification

**Figure 4:**
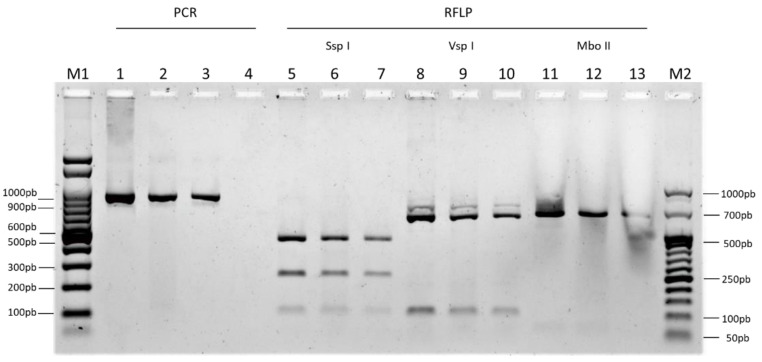
PCR-RFLP for *C. parvum* identification. M1: 100 bp marker; 1, 5, 8, 11: plasmid DNA positive controls; 2, 6, 9, 12: genomic DNA positive controls; 3, 7, 10, 13: calf inoculum; 4: negative control; M2: 50 bp marker.

One newborn calf was experimentally infected with 50 million purified oocysts from a naturally infected calf to produce the oocyst inoculum required for subsequent studies (see Section 2). Four days after inoculation, the calf developed diarrhea. Once oocyst shedding was confirmed, the fecal content was collected for further oocyst purification and species characterization by PCR-RFLP. After Ssp I, VspI, and MboII digestion of PCR amplicons, the characteristic band pattern allowed us to identify the isolated oocysts as *C. parvum* (Figure 4). Then, a second calf was challenged using 6 million purified oocysts diluted in saline solution to confirm that the inoculum was able to induce *Cryptosporidium* infection and disease. Microbiological controls (presence of rotavirus and/or coronavirus as tested by ELISA) and quantification by qPCR and microscopy of the inoculum were performed to verify its identity. Twenty milliliters of a solution containing 6 million purified oocysts was used for the inoculation of calves in the efficacy experiment.

### 3.4. Egg Powder Enriched with Anti-C. parvum P23 IgY Reduced Severe Diarrhea and Oocyst Shedding in Experimentally Challenged Calves

**Figure 5:**
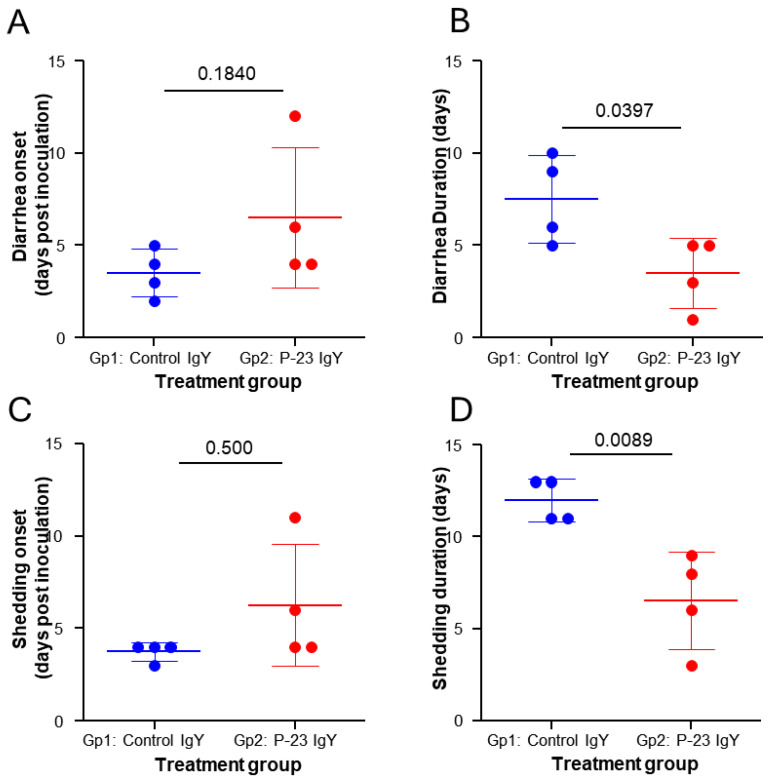
Average onset and duration of diarrhea and oocyst shedding, respectively, in calves from the control group (Gp 1) vs. the IgY-treated group (Gp 2). (**A**) Diarrhea onset. (**B**) Diarrhea duration. (**C**) Oocyst shedding. (**D**) Oocyst duration. Oocyst shedding was estimated by qPCR using the 18S rRNA gene as target. The horizontal bars represent the mean of each group for the respective clinical parameter. Vertical bars represent the standard deviation of the respective mean. Horizontal black bars and numbers represent the *p*-value of the statistical comparison.

**Figure 6:**
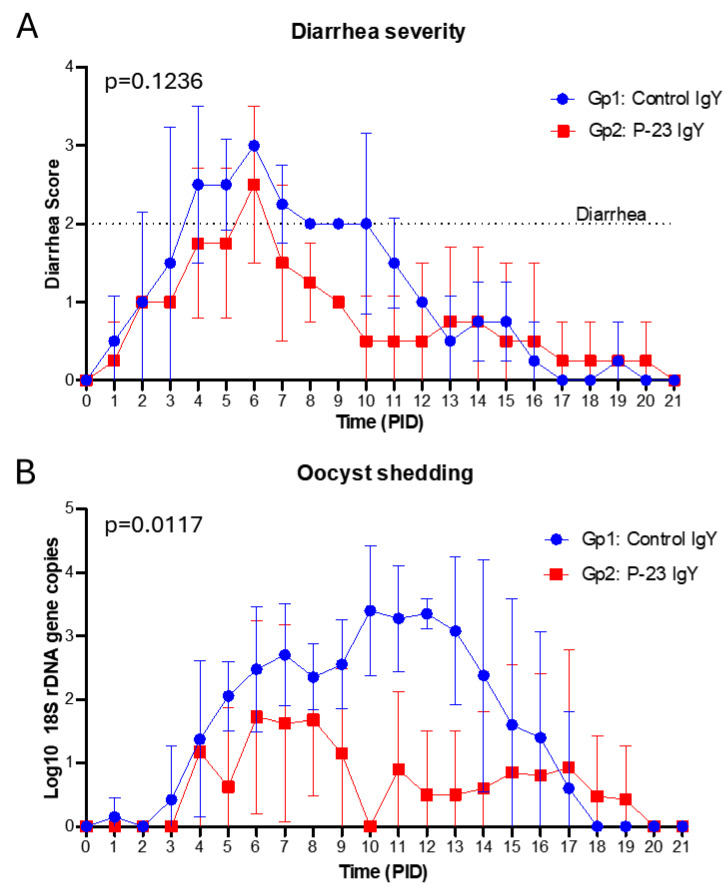
The average severity of diarrhea and amount of oocysts shed by calves in the control group (Gp 1) vs. the anti-p23 IgY-treated group (Gp 2). (**A**) The severity of diarrhea is measured as area under the curve (AUC) from fecal samples scored on a scale from 0 to 3, of which scores 2 and 3 are considered diarrhea; (**B**) Oocyst shedding is measured as the AUC of 18S rRNA gene copies as determined by qPCR.

An efficacy study evaluated whether the egg powder enriched in anti-p23 IgY could prevent or reduce *C. parvum* diarrhea in newborn calves. As detailed in Section 2.5.2, eight newborn calves were fed with 2 L of commercial colostrum reconstituted according to the manufacturer’s instructions within the first 12 h of life. The colostrum had a p23-specific IgG titer of 16,384. After colostrum intake, calves were randomly assigned to one of the following feeding groups. Group 1 (Gp1): calves fed with milk without supplementation (n = 4); group 2 (Gp2): calves fed with 2 L of milk supplemented with 20 g of egg powder enriched with anti-p23 IgY (final p23-specific IgY ELISA determined titer in milk of 256), twice daily for 14 days (n = 4). All animals were orally challenged with 6 million viable oocysts (PID 0, Post Inoculation Day 0) at 48–72 h after birth. Clinical parameters of onset, duration, and severity of diarrhea and onset, duration, and quantity of oocyst-shedding are shown in Table 1. Statistical power for all the analyzed parameters was calculated and resulted in a strong power value equal to or greater than 0.80 (see Supplementary Materials Part S1). Following inoculation with *C. parvum*, all calves developed diarrhea, with the average onset occurring at PID 3.5 in the control group and at PID 6.5 in the IgY-treated group (*p* = 0.1840, statistical power of 0.76) (Table 1 and Figure 5A). Additionally, the severity of diarrhea observed in the treated calves was less pronounced than that observed in the control group (*p* = 0.1236, statistical power of 0.80) (Table 1, Figure 6A). However, both these differences were not statistically significant. In contrast, the administration of milk supplemented with IgY resulted in a significantly shorter duration of diarrheic episodes (*p* = 0.0397, statistical power of 0.88) when compared with the control group (3.50 vs. 7.50 days, respectively) (Table 1 and Figure 5B).

The severity of diarrhea was measured as the area under the curve (AUC) of fecal scores of calves (0 to 3, of which 2 and 3 were considered diarrhea) during the 14 days of treatment. The quantity of oocyst shedding was measured as the AUC of genome copies (18S rRNA gene). PID: post-inoculation days. Means in the same column that have different superscript letters (A and B) indicate a statistically significant difference (Student’s t-test was applied to all clinical parameters).

Oocyst shedding was measured by qPCR targeting the 18S rRNA gene, as detailed in Section 2. All animals showed signs of infection and shed oocysts starting at PID 3.75 in the control animals and at PID 6.25 in treated animals (*p* = 0.500, statistical power of 0.76) (Table 1 and Figure 5C). However, the p23-specific IgY group of calves (Gp2) showed a significantly shorter (*p* = 0.0089, statistical power of 0.95) shedding duration of oocysts compared to the control group (6.50 vs. 12, respectively) (Table 1, Figure 5D and Figure 6B). In addition, the quantity of shed oocysts (AUC) of the treated group was significantly reduced (*p* = 0.0117, statistical power of 0.94) when compared with the group of animals that received non-supplemented milk (Table 1 and Figure 6B). Neither rotavirus nor coronavirus were detected in the calves’ feces.

**Figure 7:**
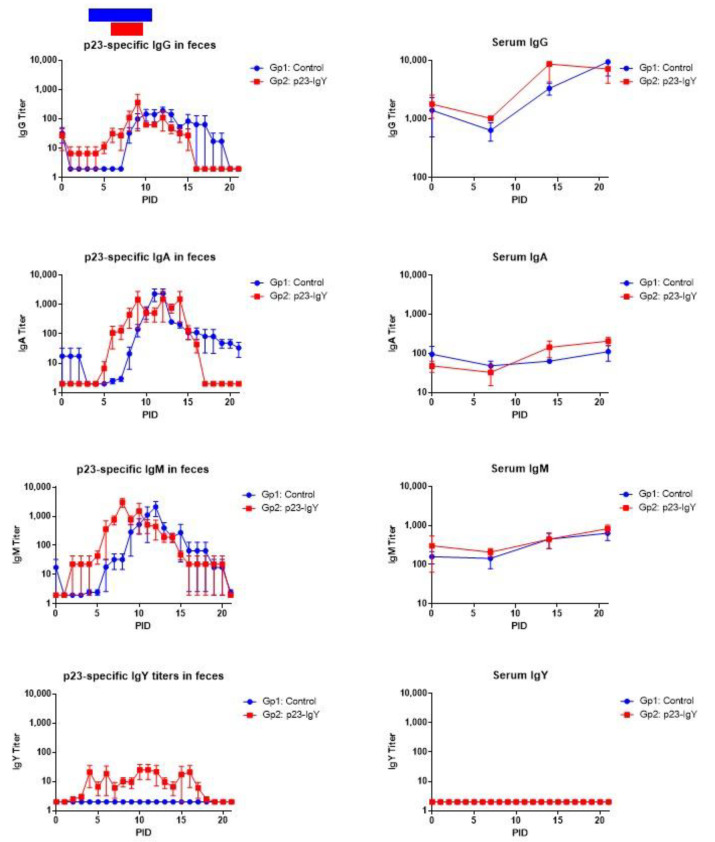
Geometric mean titers (GMT) of *C. parvum* p23-isotype-specific antibodies in fecal samples and sera from calves in the control group (Gp1) and p23-IgY-treated group (Gp2) as determined for 21 post-inoculation days (PID). IgG, IgA, IgM, and IgY were measured by indirect ELISA. Vertical bars represent the standard deviation of the mean of each day. Horizontal bars represent the average duration of diarrhea.

The isotype-specific antibody response to *C. parvum* infection was evaluated in the feces and sera of calves from both groups (Figure 7). Fecal IgM and IgA responses were found to be associated with parasite clearance and the resolution of diarrhea in both groups (Figure 7). The peaks of fecal IgA responses (~4096) were detected at PID 9 in the p23-specific IgY-treated group and at PID 11 in the control group. The fecal IgM responses (~4096) peaked earlier at PID 7 in the p23-specific IgY group compared to PID 12 in the control group. Low levels of IgG were detected in the fecal samples, with a peak at PID 9 in the p23-specific IgY group (~256) and a peak at PID 10 in the control group (~128). IgY Abs in fecal samples were detected intermittently and in low titers in treated calves until three days after the end of the treatment. In serum, IgA, IgM, and IgG Ab responses were of similar magnitude in both treated and control groups (Figure 7). However, the peak of p23-specific serum IgG was at PID 15 in the IgY-treated group while at PID 21 in the control group. There was no detection of IgY Abs in serum from treated calves, confirming no transfer of IgY from the gut into the bloodstream.

Finally, we evaluated the clinical parameter hyperthermia. To this end, rectal temperature was measured daily in all calves participating in this study. No hyperthermia was detected in any calf of the treated and control groups.

## 4. Discussion

We evaluated the efficacy of p23-specific IgY treatment in neonatal calves experimentally infected with *C. parvum* to reduce clinical symptoms and oocyst shedding. For this purpose, we generated an experimental vaccine by using a recombinant APCH-p23 immunogen to produce a p23-specific IgY product. Bioinnovo S.A, a Start-up company from Argentina, used and validated APCH as an auxiliary component in a biotechnological vaccine to control bovine viral diarrhea (BVD) in cattle [25], which showed an outstanding performance in beef and dairy herds in Argentina [30]. APCH is a single-chain Ab directed to the MHC-II antigen epitope and has been designated as a potent immunomodulating molecule improving both humoral and cellular immune responses in immunized animals as it targets the antigen to the APCs (antigen-presenting cells) [18]. The passive vaccination procedure tested in this study is based on the p23 antigen, which belongs to a family of 23–27 kDa surface antigens that are expressed in different parasite stages during the life cycle of *C. parvum*. Immunostaining analysis using indirect fluorescent antibodies showed that p23 can be recognized on the surface of oocysts [12]. Importantly, as a recombinant antigen, p23 has been tested in vaccination trials of pregnant heifers and shown to generate hyperimmune colostrum, mitigating severe diarrhea and oocyst shedding in neonatal calves [15,16]. Furthermore, it has been demonstrated that p23 has neutralization-sensitive epitopes [31,32,33]. Additionally, other surface antigens such as gp40 and gp900 have been demonstrated to contain neutralization-sensitive epitopes that represent targets of vaccine or monoclonal antibodies for the development of effective control strategies [20,34].

The production of antigen-specific IgY has been applied to a wide range of pathogens, and its production in chickens is a more sustainable and economic way of generating polyclonal antibodies for passive immunization or immunotherapeutic procedures [34,35,36]. We previously demonstrated that rotavirus A and bovine coronavirus-specific IgY antibodies reduced or even prevented clinical signs of each viral infection in newborn calves [21,22]. Recently, we demonstrated that the enteropathogen *C. parvum* is the major cause of neonatal calf diarrhea in Argentina, motivating us to test the efficacy of *C. parvum* antigen-specific IgY for the treatment of this ubiquitous yet neglected parasitic infection [7]. In several studies, it has been shown that IgY Abs produced against oocyst lysate or recombinant p23 or gp60 of *C. parvum* could decrease oocyst excretion in mice [12,37,38]. This urged us to test whether antigen-specific IgY is effective in the prevention and/or treatment of diarrheic calves. In our approach, immunity is conferred by p23-specific IgY produced in chicken as compared to the immunity conferred to calves via p23-specific IgG in colostrum generated after immunizing pregnant heifers. Importantly, the p23-specific IgY product allows high flexibility in the number of applied doses and the application scheme, and besides conferring passive immunity, it may also be applied in an immunotherapeutic procedure to cure calves already diarrheic. In the present study, calves were inoculated with six million oocysts at 48–72 h of life. This amount is comparable to the inoculation with ten million oocysts at 12 h of life that had been applied by Perryman et al. and Askari et al. [15,16] or the one million from the study of Bhalchandra and collaborators [39]. However, other authors used a reduced number of oocysts (e.g., 1000) to infect calves for vaccine efficacy studies [20], which is in line with Zambriski and collaborators, who observed that 50 oocysts are sufficient to consistently produce diarrhea and oocyst shedding [40]. However, it needs to be considered that *Cryptosporidium* is a highly endemic parasite on dairy farms in Argentina with the potential for high natural infection doses. The present study evaluates a passive treatment administered for 14 consecutive days so that antibodies are continuously present in the gut, as opposed to a vaccine efficacy study where the dam is vaccinated, and the calf is passively protected by a single ingestion of hyperimmune colostrum. Based on these considerations, we hypothesized that the prolonged presence of p23-specific IgY in the gut lumen would be able to attenuate the clinical disease of severe cryptosporidiosis in calves, even when challenged with a very high number of oocysts.

After challenge, we observed that control calves shed *C. parvum* oocysts and developed diarrhea for an average of 7.5 days starting at 3.5 days post-inoculation, which is similar to what has been shown in other studies when infected calves were fed with commercial colostrum [28,40]. It needs to be highlighted that in this efficacy study, calves were fed with commercially available colostrum with a p23-specific IgG titer of 16384. This titer is rather low, considering that IgG1 is concentrated in the colostrum to confer an efficient immune transfer to the calf. However, information on colostrum antibodies and their association with *C. parvum*-induced diarrhea is scarce. Some authors report that the level of parasite-specific antibodies in colostrum correlates with the prevention of diarrhea caused by *C. parvum* [41], but the minimum titer of parasite-specific Abs in the serum of calves after colostrum intake which is needed to prevent *C. parvum* diarrhea is still unknown. Thus, improving the colostrum quality by vaccination against *C. parvum* and supplementing the milk with parasite-specific IgY during the susceptible immunological window, when colostrum Abs decrease and host immune response begins to develop, has the potential to minimize or even prevent clinical signs of *C. parvum* infection under field conditions.

In the present study, calves fed with heterologous IgY with a high p23-specific IgY titer of 256 showed a delay in the onset of diarrhea and oocyst shedding compared to control animals, although this delay was not significant. In addition, the severity of diarrhea was shown to be non-significantly reduced in treated vs. non-treated control. In contrast, the clinical episodes, such as duration of diarrhea and oocyst shedding in IgY-treated calves, were significantly shorter in duration. Furthermore, IgY-treated calves shed significantly fewer oocysts (measured as the AUC of 18S rRNA gene copies) compared to the non-treated control group. This finding is consistent with that reported by Omidian et al. (2014), in which *C. parvum* p23-specific IgY was administered to immunosuppressed mice that had been inoculated with ten thousand oocysts resulting in a significantly lower oocyst excretion [12]. Additionally, Kobayashi and collaborators produced IgY by immunizing hens with an oocyst preparation and tested its efficacy in a mouse model. Treated mice showed shedding of a lower number of oocysts compared to control animals following inoculation with one thousand oocysts [38]. On the other hand, monoclonal Abs (mAb) directed to different epitopes of the infective *C. parvum* oocyst comprising anti-GP25-200, anti-P23, and anti-CSL proteins were tested in a mouse model by Schaefer and colleagues [31]. A pool of these three monoclonal antibodies (75 µg of each mAb per dose) was demonstrated to reduce the level of intestinal infection by 86% in mice challenged with thirty thousand oocysts, as measured by histopathological scores [31]. These results demonstrate that antibodies directed to different epitopes of the parasite can reduce oocyst shedding in mice, which suggests that they are also effective in the mitigation of diarrhea in calves.

It needs to be highlighted that the number of animals used in this study was estimated following animal welfare protocols and is supported with an acceptable statistical power for a preliminary efficacy study. Considering the observed positive effect of the p23-IgY treatment, a field trial with a higher number of animals and natural exposure to *C. parvum* will be carried out to substantiate the effectiveness of passive p23-IgY treatment. In addition, in future studies, a higher administration dose of 40 g with a final p23-specific IgY titer in milk of higher than 256 will be applied to test the hypothesis that this further improves the efficacy of the treatment.

In the present study, all calves from the control and treated groups received preventive antibiotic treatment to avoid undesirable infections of pathogenic bacteria. Recently, it has been shown that the decrease in short-chain fatty acids in the gut is associated with an increased *C. parvum* infection in mice [42,43]. In this context, it is noteworthy that the presence of some bacteria, such as *Megasphera* spp., synthesize short-chain fatty acids [43]. Although it is known that antibiotic treatment changes the gut microbiota and may affect *C. parvum* infection and disease, antibiotic treatment was applied to both groups and should, therefore, not influence the experimental result.

The study of the specific immune response of calves to *C. parvum* infection revealed that mucosal IgA and IgM responses were associated with clearance of the parasite infection in both study groups. Noteworthy, in treated calves, the level of this class of antibodies rises and peaks at an earlier PID than in control animals. Although these findings are similar to those observed by Wyatt et al., where mucosal antibodies were detected after diarrhea resolution, they seem to be counterintuitive [44]. Currently, we do not have an explanation for this observation since both groups of animals had a similar onset of diarrhea and oocyst shedding. Furthermore, Peeters et al. described that the immune response after experimental and natural infection showed rising IgM levels in feces from PID 5 on, with a peak at PID 14, and IgA levels in feces rose between PID 7 and PID 14, which both coincided with a decreasing excretion of oocysts [45]. Interestingly, IgG levels in feces rose slightly during oocyst shedding yet disappeared 3 weeks after post-infection [45]. In the same study, anti-*C. parvum* IgG levels in sera rose till oocyst excretion reached its maximum, whereas anti-*C. parvum* IgA levels in sera peaked later than those of IgA levels in feces [45]. Importantly, the immune response against *C. parvum* in calves is not fully understood; it has been reported that the innate immune response plays a key role in parasitic clearance in which IFNγ produced by NK cells and macrophages had a protective effect [46,47]. The inability of IFNγ gene-knockout mice to control *C. parvum* infection was associated with reduced recruitment of macrophages and T cells to the lamina propria, suggesting this cytokine and immune cells play an important role in the control of the infection in mice [48]. Experimental infections of mice or humans with *Cryptosporidium* induce mucosal IgG and IgA antibodies, which may correlate with the quantity of oocyst shedding in humans [49]. In the presented study, similar IgG, IgA, and IgM responses were found in sera of both study groups after infection with *C. parvum*. Kaushik and collaborators observed a higher IgG and IgA titer in *Cryptosporidium*-infected human subjects compared to those that were uninfected [50]. Importantly, a recent study that analyzed the protective impact of *Cryptosporidium*-specific Ab in a cohort of Bangladesh children has found that Ab responses to antigens p23 and gp60 correlated with protection against reinfection [51]. Finally, p23-specific IgY was found to be functional in fecal samples as tested during the complete treatment period, suggesting that IgY passes through the entire gastrointestinal tract without denaturation or functional neutralization, as has also been observed in previous studies [21,22]. Additional studies are needed to elucidate the mechanism of neutralization of p23-specific IgY. As mentioned before, mucosal immunity is highly important in *C. parvum* infection, where p23 mucosal antibodies (mostly IgA) are produced, as was observed by Wyatt and collaborators [52,53]. It has also been reported that after ingestion of egg yolk, molecules positively modulate the mucosal immune response, preventing pathogen entry [21,54].

Recently, a vaccination for pregnant heifers based on recombinant gp40 was tested and shown to significantly reduce the incidence and severity of diarrhea in *C. parvum*-infected calves [20]. This vaccine is available in European countries but not in Argentina. The presented passive immunization strategy of *C. parvum*-infected calves using antigen-specific IgY resulted in a significantly reduced duration of diarrhea and oocyst excretion and has the advantage of treatment flexibility, as calves can still be treated in case pregnant heifers had not been previously immunized. Passive immunization using p23-specific IgY has the potential to reduce oocyst contamination in farms, thereby minimizing the risk of parasite transmission between calves and reducing the public risk of human infection due to environmental contamination. We have developed a sustainable, non-antibiotic treatment to control diarrhea in newborn calves caused by *C. parvum*, which shows promising efficacy for controlling *C. parvum*-associated diarrhea. Field trials under natural conditions are required to substantiate the efficacy of this development.

## 5. Conclusions

We show that *Cryptosporidium parvum*-specific IgY treatment is a promising passive immunization strategy to control *C. parvum*-associated diarrhea. The administration of p23-specific IgY in milk for 14 days significantly reduced the duration of diarrhea and oocyst shedding. The recombinantly expressed APCH-p23 is an excellent candidate to produce anti-p23 IgY antibodies resulting in high titers in hens. The immune response against *C. parvum* was similar in control and treated animals, and fecal IgA and IgM levels were associated with parasitic clearance. This is the first study that evaluates the efficacy of an IgY-based product to control *C. parvum* diarrhea in neonatal calves under controlled conditions.

## Figures and Tables

**Table 1:** Clinical parameters of onset, duration, and severity of diarrhea and oocyst shedding of calves experimentally challenged with *C. parvum* oocysts. Different superscript letters indicate a statistically significant difference.

			Diarrhea			Oocyst Shedding		
Treatment Group	Group (Gp)	n	Onset (PID)	Duration (Days)	Severity (AUC)	Onset (PID)	Duration (Days)	Quantity (AUC)
Control	1	4	3.50	7.50 ^A^	24.25	3.75	12.00 ^A^	33.45 ^A^
P23-IgY	2	4	6.50	3.50 ^B^	17.00	6.25	6.50 ^B^	14.25 ^B^
*p*-value			0.1840	0.0397	0.1236	0.500	0.0089	0.0117

## Data Availability

Data are contained within the article and Supplementary Materials.

## Supplementary Materials

The following supporting information can be downloaded at: https://www.mdpi.com/article/10.3390/vaccines13020162/s1, Figure S1: Statistical power calculations of each analyzed parameter of infection and disease caused by *C. parvum*; Figure S2: ELISA assay to confirm specificity of IgY to p23 in the oocyst.

## References

[1] Ryan U.M., Feng Y., Fayer R., Xiao L. (2021). Taxonomy and Molecular Epidemiology of Cryptosporidium and Giardia—A 50 Year Perspective (1971–2021). Int. J. Parasitol..

[2] Santín M., Trout J.M., Xiao L., Zhou L., Greiner E., Fayer R. (2004). Prevalence and Age-Related Variation of Cryptosporidium Species and Genotypes in Dairy Calves. Vet. Parasitol..

[3] Khalil I.A., Troeger C., Rao P.C., Blacker B.F., Brown A., Brewer T.G., Colombara D.V., De Hostos E.L., Engmann C., Guerrant R.L. (2018). Morbidity, Mortality, and Long-Term Consequences Associated with Diarrhoea from *Cryptosporidium* Infection in Children Younger than 5 Years: A Meta-Analyses Study. Lancet Glob. Health.

[4] Korpe P.S., Valencia C., Haque R., Mahfuz M., McGrath M., Houpt E., Kosek M., McCormick B.J.J., Penataro Yori P., Babji S. (2018). Epidemiology and Risk Factors for Cryptosporidiosis in Children From 8 Low-Income Sites: Results From the MAL-ED Study. Clin. Infect. Dis. Off. Publ. Infect. Dis. Soc. Am..

[5] Chen Y., Huang J., Qin H., Wang L., Li J., Zhang L. (2023). Cryptosporidium Parvum and Gp60 Genotype Prevalence in Dairy Calves Worldwide: A Systematic Review and Meta-Analysis. Acta Trop..

[6] Lombardelli J.A., Tomazic M.L., Schnittger L., Tiranti K.I. (2019). Prevalence of *Cryptosporidium parvum* in Dairy Calves and GP60 Subtyping of Diarrheic Calves in Central Argentina. Parasitol. Res..

[7] Garro C.J., Morici G.E., Tomazic M.L., Vilte D., Encinas M., Vega C., Bok M., Parreño V., Schnittger L. (2021). Occurrence of Cryptosporidium and Other Enteropathogens and Their Association with Diarrhea in Dairy Calves of Buenos Aires Province, Argentina. Vet. Parasitol. Reg. Stud. Rep..

[8] de Alba P., Garro C., Florin-Christensen M., Schnittger L. (2023). Prevalence, Risk Factors and Molecular Epidemiology of Neonatal Cryptosporidiosis in Calves: The Argentine Perspective. Curr. Res. Parasitol. Vector-Borne Dis..

[9] Askenaizer D., Meyers R.A. (2003). Drinking Water Quality and Treatment. Encyclopedia of Physical Science and Technology.

[10] Thompson RCAFayer R., Xiao L. (2008). Cryptosporidium and Cryptosporidiosis. Parasit. Vectors.

[11] Bouzid M., Hunter P.R., Chalmers R.M., Tyler K.M. (2013). Cryptosporidium Pathogenicity and Virulence. Clin. Microbiol. Rev..

[12] Omidian Z., Ebrahimzadeh E., Shahbazi P., Asghari Z., Shayan P. (2014). Application of Recombinant Cryptosporidium Parvum P23 for Isolation and Prevention. Parasitol. Res..

[13] Arrowood M.J., Sterling C.R., Healey M.C. (1991). Immunofluorescent Microscopical Visualization of Trails Left by Gliding Cryptosporidium Parvum Sporozoites. J. Parasitol..

[14] Boulter-Bitzer J.I., Lee H., Trevors J.T. (2007). Molecular Targets for Detection and Immunotherapy in Cryptosporidium Parvum. Biotechnol. Adv..

[15] Perryman L.E., Kapil S.J., Jones M.L., Hunt E.L. (1999). Protection of Calves against Cryptosporidiosis with Immune Bovine Colostrum Induced by a Cryptosporidium Parvum Recombinant Protein. Vaccine.

[16] Askari N., Shayan P., Mokhber-Dezfouli M.R., Ebrahimzadeh E., Lotfollahzadeh S., Rostami A., Amininia N., Ragh M.J. (2016). Evaluation of Recombinant P23 Protein as a Vaccine for Passive Immunization of Newborn Calves against Cryptosporidium Parvum. Parasite Immunol..

[17] Shirafuji H., Xuan X., Kimata I., Takashima Y., Fukumoto S., Otsuka H., Nagasawa H., Suzuki H. (2005). Expression of P23 of Cryptosporidium Parvum in Toxoplasma Gondii and Evaluation of Its Protective Effects. J. Parasitol..

[18] Gil F., Pérez-Filgueira M., Barderas M.G., Pastor-Vargas C., Alonso C., Vivanco F., Escribano J.M. (2011). Targeting Antigens to an Invariant Epitope of the MHC Class II DR Molecule Potentiates the Immune Response to Subunit Vaccines. Virus Res..

[19] Drugmand J.-C., Schneider Y.-J., Agathos S.N. (2012). Insect Cells as Factories for Biomanufacturing. Biotechnol. Adv..

[20] Timmermans M., Hubers W., Schroer D., Gevers K., Segers R.P.A.M., Niessen R., van Roosmalen M.H. (2024). The First Commercially Approved Efficacious Cryptosporidium Vaccine Protecting New-Born Calves from Severe Diarrhea. Vet. Vaccine.

[21] Vega C., Bok M., Chacana P., Saif L., Fernandez F., Parreno V. (2011). Egg Yolk IgY: Protection against Rotavirus Induced Diarrhea and Modulatory Effect on the Systemic and Mucosal Antibody Responses in Newborn Calves. Vet. Immunol. Immunopathol..

[22] Bok M., Vega C.G., Castells M., Colina R., Wigdorovitz A., Parreño V. (2023). Development of an IgY-Based Treatment to Control Bovine Coronavirus Diarrhea in Dairy Calves. Viruses.

[23] Agurto-Arteaga A., Poma-Acevedo A., Rios-Matos D., Choque-Guevara R., Montesinos-Millán R., Montalván Á., Isasi-Rivas G., Cauna-Orocollo Y., Cauti-Mendoza M.d.G., Pérez-Martínez N. (2022). Preclinical Assessment of IgY Antibodies Against Recombinant SARS-CoV-2 RBD Protein for Prophylaxis and Post-Infection Treatment of COVID-19. Front. Immunol..

[24] Vega C.G., Bok M., Ebinger M., Rocha L.A., Rivolta A.A., González Thomas V., Muntadas P., D’Aloia R., Pinto V., Parreño V. (2020). A New Passive Immune Strategy Based on IgY Antibodies as a Key Element to Control Neonatal Calf Diarrhea in Dairy Farms. BMC Vet. Res..

[25] Bellido D., Baztarrica J., Rocha L., Pecora A., Acosta M., Escribano J.M., Parreño V., Wigdorovitz A. (2021). A Novel MHC-II Targeted BVDV Subunit Vaccine Induces a Neutralizing Immunological Response in Guinea Pigs and Cattle. Transbound. Emerg. Dis..

[26] Arrowood M.J., Mead J.R., Arrowood M.J. (2020). Cryptosporidium Oocyst Purification Using Discontinuous Gradient Centrifugation. Cryptosporidium: Methods and Protocols.

[27] Kimbell L.M., Miller D.L., Chavez W., Altman N. (1999). Molecular Analysis of the 18S RRNA Gene of Cryptosporidium Serpentis in a Wild-Caught Corn Snake (Elaphe Guttata Guttata) and a Five-Species Restriction Fragment Length Polymorphism- Based Assay That Can Additionally Discern C. Parvum from C. Wrairi. Appl. Environ. Microbiol..

[28] Riggs M.W., Schaefer D.A., Mead J.R., Arrowood M.J. (2020). Calf Clinical Model of Cryptosporidiosis for Efficacy Evaluation of Therapeutics. Cryptosporidium: Methods and Protocols.

[29] Jothikumar N., da Silva A.J., Moura I., Qvarnstrom Y., Hill V.R. (2008). Detection and Differentiation of Cryptosporidium Hominis and Cryptosporidium Parvum by Dual TaqMan Assays. J. Med. Microbiol..

[30] Bellido D., Gumina E.R., Rodríguez Senes G.J., Chiariotti F.M., Audrito M., Sueldo P.M., Sueldo G.M., Wigdorovitz A. (2024). First Evaluation of the Impact of a Targeted Subunit Vaccine against Bovine Viral Diarrhea Virus in Feedlot Cattle. Transl. Anim. Sci..

[31] Schaefer D.A., Auerbach-Dixon B.A., Riggs M.W. (2000). Characterization and Formulation of Multiple Epitope-Specific Neutralizing Monoclonal Antibodies for Passive Immunization against Cryptosporidiosis. Infect. Immun..

[32] Perryman L.E., Jasmer D.P., Riggs M.W., Bohnet S.G., McGuire T.C., Arrowood M.J. (1996). A Cloned Gene of Cryptosporidium Parvum Encodes Neutralization-Sensitive Epitopes. Mol. Biochem. Parasitol..

[33] Lemieux M.W., Sonzogni-Desautels K., Ndao M. (2017). Lessons Learned from Protective Immune Responses to Optimize Vaccines against Cryptosporidiosis. Pathogens.

[34] Schade R., Calzado E.G., Sarmiento R., Chacana P.A., Porankiewicz-Asplund J., Terzolo H.R. (2005). Chicken Egg Yolk Antibodies (IgY-Technology): A Review of Progress in Production and Use in Research and Human and Veterinary Medicine. Altern. Lab. Anim..

[35] Karachaliou C.-E., Vassilakopoulou V., Livaniou E. (2021). IgY Technology: Methods for Developing and Evaluating Avian Immunoglobulins for the in Vitro Detection of Biomolecules. World J. Methodol..

[36] Yakhkeshi S., Wu R., Chelliappan B., Zhang X. (2022). Trends in Industrialization and Commercialization of IgY Technology. Front. Immunol..

[37] Kobayashi C., Yokoyama H., Nguyen S.V., Kodama Y., Kimata T., Izeki M. (2004). Effect of Egg Yolk Antibody on Experimental Cryptosporidium Parvum Infection in Scid Mice. Vaccine.

[38] Miura V.C., Aoki S.M., Peitl P., Pires L.C., Dalmagro P., Nakamura A.A., Meireles M.V. (2017). Evaluation of Recombinant *Cryptosporidium hominis* GP60 Protein and Anti-GP60 Chicken Polyclonal IgY for Research and Diagnostic Purposes. Rev. Bras. Parasitol. Veterinária.

[39] Bhalchandra S., Gevers K., Heimburg-Molinaro J., van Roosmalen M., Coppens I., Cummings R.D., Ward H.D. (2023). Identification of the Glycopeptide Epitope Recognized by a Protective *Cryptosporidium* Monoclonal Antibody. Infect. Immun..

[40] Zambriski J.A., Nydam D.V., Wilcox Z.J., Bowman D.D., Mohammed H.O., Liotta J.L. (2013). Cryptosporidium Parvum: Determination of ID₅₀ and the Dose-Response Relationship in Experimentally Challenged Dairy Calves. Vet. Parasitol..

[41] Lefkaditis M., Mpairamoglou R., Sossidou A., Spanoudis K., Tsakiroglou M., Györke A. (2020). Importance of Colostrum IgG Antibodies Level for Prevention of Infection with Cryptosporidium Parvum in Neonatal Dairy Calves. Prev. Vet. Med..

[42] Xin H., Ma T., Xu Y., Chen G., Chen Y., Villot C., Renaud D.L., Steele M.A., Guan L.L. (2021). Characterization of Fecal Branched-Chain Fatty Acid Profiles and Their Associations with Fecal Microbiota in Diarrheic and Healthy Dairy Calves. J. Dairy Sci..

[43] Morita Y., Yachida M., Tokimitsu K., Itoh M. (2024). Analysis of Gut Microbiota with Cryptosporidiosis Based on Fecal Condition in Neonatal Dairy Calves on a Farm in Japan. JDS Commun..

[44] Wyatt C.R. (2000). Cryptosporidium Parvum and Mucosal Immunity in Neonatal Cattle. Anim. Health Res. Rev..

[45] Peeters J.E., Villacorta I., Vanopdenbosch E., Vandergheynst D., Naciri M., Ares-Mazás E., Yvoré P. (1992). Cryptosporidium Parvum in Calves: Kinetics and Immunoblot Analysis of Specific Serum and Local Antibody Responses (Immunoglobulin A [IgA], IgG, and IgM) after Natural and Experimental Infections. Infect. Immun..

[46] Barakat F.M., McDonald V., Di Santo J.P., Korbel D.S. (2009). Roles for NK Cells and an NK Cell-Independent Source of Intestinal Gamma Interferon for Innate Immunity to Cryptosporidium Parvum Infection. Infect. Immun..

[47] Lacroix-Lamandé S., Mancassola R., Naciri M., Laurent F. (2002). Role of Gamma Interferon in Chemokine Expression in the Ileum of Mice and in a Murine Intestinal Epithelial Cell Line after Cryptosporidium Parvum Infection. Infect. Immun..

[48] Thomson S., Hamilton C.A., Hope J.C., Katzer F., Mabbott N.A., Morrison L.J., Innes E.A. (2017). Bovine Cryptosporidiosis: Impact, Host-Parasite Interaction and Control Strategies. Vet. Res..

[49] Pardy R.D., Wallbank B.A., Striepen B., Hunter C.A. (2024). Immunity to Cryptosporidium: Insights into Principles of Enteric Responses to Infection. Nat. Rev. Immunol..

[50] Kaushik K., Khurana S., Wanchu A., Malla N. (2009). Serum Immunoglobulin G, M and A Response to Cryptosporidium Parvum in Cryptosporidium-HIV Co-Infected Patients. BMC Infect. Dis..

[51] Gilchrist C.A., Campo J.J., Pablo J.V., Ma J.Z., Teng A., Oberai A., Shandling A.D., Alam M., Kabir M., Faruque A.S.G. (2023). Specific Cryptosporidium Antigens Associate with Reinfection Immunity and Protection from Cryptosporidiosis. J. Clin. Investig..

[52] Wyatt C.R., Perryman L.E. (2000). Detection of Mucosally Delivered Antibody to Cryptosporidium Parvum P23 in Infected Calves. Ann. N. Y. Acad. Sci..

[53] Wyatt C.R., Brackett E.J., Mason P.H., Savidge J., Perryman L.E. (2000). Excretion Patterns of Mucosally Delivered Antibodies to P23 in Cryptosporidium Parvum Infected Calves. Vet. Immunol. Immunopathol..

[54] Nelson R., Katayama S., Mine Y., Duarte J., Matar C. (2007). Immunomodulating Effects of Egg Yolk Low Lipid Peptic Digests in a Murine Model. Food Agric. Immunol..

