# Supplementary material for: P23-Specific IgY Significantly Reduces Diarrhea and Oocyst Shedding in Calves Experimentally Infected with Cryptosporidium parvum
Source: Vaccines (Basel). 2025 Feb 7;13(2):162. doi: 10.3390/vaccines13020162 (PMC11860195; doi:10.3390/vaccines13020162)
Supplement: Supplementary file 1 [file vaccines-13-00162-s001.zip › vaccines-3394233-supplementary.pdf]

Supplementary Material 1

Statistical power calculations of each analyzed parameter of infection and disease caused by *C. parvum*. A power value equal to or greater than 0.80 is considered a good power value.

1- Diarrhea Severity

Control	P-23-IgY
Mean control=24.25	Mean P-23 IgY=17
SD control=5.85	SD P-23 IgY=5.59

t tests – Means: Difference between two independent means (two groups)

**Analysis:**	Compromise: Compute implied α & power		
**Input:**	Tail(s)	=	One
	Effect size d	=	1.2671553
	β/α ratio	=	1
	Sample size group 1	=	4
	Sample size group 2	=	4
**Output:**	Noncentrality parameter δ	=	1.7920282
	Critical t	=	0.9341971
	Df	=	6
	α err prob	=	0.1931281
	β err prob	=	0.1931281
	**Power (1−β err prob)**	**=**	**0.8068719**

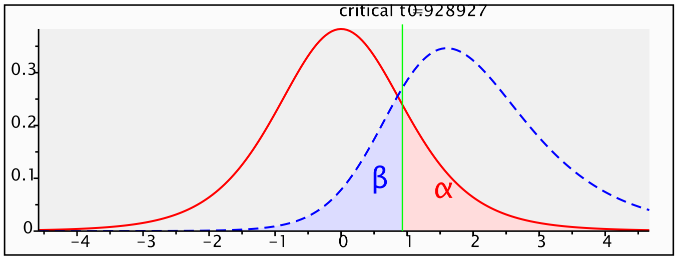

Distribution Plot of Diarrhea Severity

2- Diarrhea Duration

Control	P-23-IgY
Mean control=7.5	Mean P-23-IgY=3.5
SD control=2.39	SD P-23-IgY=1.915

t tests – Means: Difference between two independent means (two groups)

Analysis: Compromise: Compute implied α & power

**Input:**	Tail(s)	=	One
	Effect size d	=	1.8470948
	β/α ratio	=	1
	Sample size group 1	=	4
	Sample size group 2	=	4
**Output:**	Noncentrality parameter δ	=	2.6121865
	Critical t	=	1.3600667
	Df	=	6
	α err prob	=	0.1113448
	β err prob	=	0.1113448
	**Power (1−β err prob)**	**=**	**0.8886552**

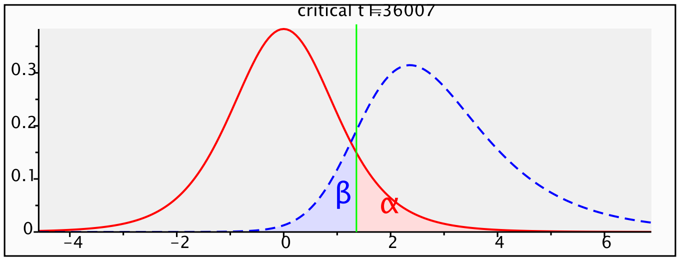

Distribution Plot of Diarrhea Duration

3- Diarrhea Onset

Control	P-23-IgY
Mean control=3.50	Mean P-23-IgY=6.50
SD control=1.29	SD P-23-IgY=3.78

t tests – Means: Difference between two independent means (two groups)

**Analysis:**	Compromise: Compute implied α & power		
**Input:**	Tail(s)	=	One
	Effect size d	=	1.0622381
	β/α ratio	=	1
	Sample size group 1	=	4
	Sample size group 2	=	4
**Output:**	Noncentrality parameter δ	=	1.5022315
	Critical t	=	0.7831896
	Df	=	6
	α err prob	=	0.2316496
	β err prob	=	0.2316496
	**Power (1−β err prob)**	**=**	**0.7683504**

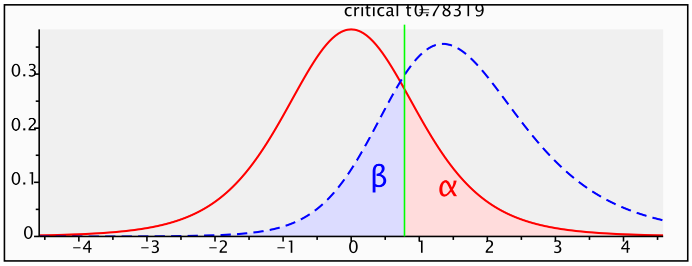

Distribution Plot of Diarrhea Onset

4- Oocyst Severity

Control	P-23-IgY
Mean control=33.45	Mean P-23-IgY=14.25
SD control=6.95	SD P-23-IgY=8.18

t tests – Means: Difference between two independent means (two groups)

**Analysis:**	Compromise: Compute implied α & power		
**Input:**	Tail(s)	=	One
	Effect size d	=	2.5296585
	β/α ratio	=	1
	Sample size group 1	=	4
	Sample size group 2	=	4
**Output:**	Noncentrality parameter δ	=	3.5774774
	Critical t	=	1.8549948
	Df	=	6
	α err prob	=	0.0565025
	β err prob	=	0.0565025
	**Power (1–β err prob)**	**=**	**0.9434975**

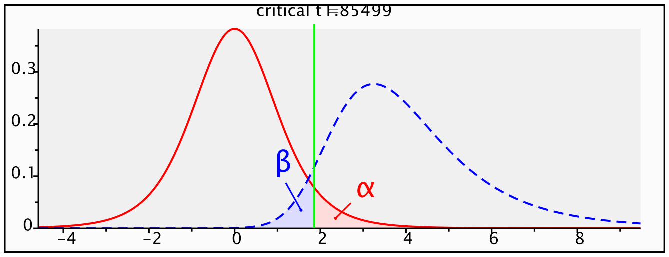

Distribution Plot of Oocyst Severity

5- Oocyst Duration

Control	P-23-IgY
Mean control=12.00	Mean P-23-IgY=6.50

SD control=1.15	SD P-23-IgY=2.64

t tests – Means: Difference between two independent means (two groups)

**Analysis:**	Compromise: Compute implied α & power		
**Input:**	Tail(s)	=	One
	Effect size d	=	2.7011296
	β/α ratio	=	1
	Sample size group 1	=	4
	Sample size group 2	=	4
**Output:**	Noncentrality parameter δ	=	3.8199741
	Critical t	=	1.9776991
	Df	=	6
	α err prob	=	0.0476637
	β err prob	=	0.0476637
	**Power (1−β err prob)**	**=**	**0.9523363**

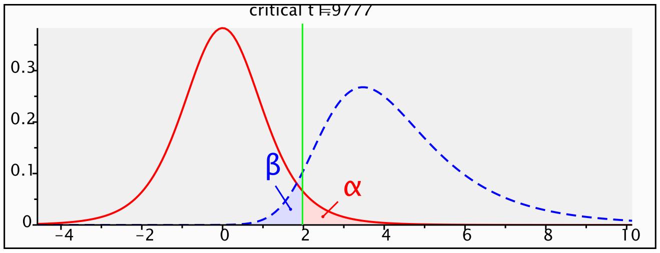

Distribution Plot of
Oocyst Duration

6- Oocyst Onset

t tests – Means: Difference between two independent means (two groups)

**Analysis:**	Compromise: Compute implied α & power		
**Input:**	Tail(s)	=	One
	Effect size d	=	1.0592840
	β/α ratio	=	1
	Sample size group 1	=	4
	Sample size group 2	=	4
**Output:**	Noncentrality parameter δ	=	1.4980538
	Critical t	=	0.7810118
	Df	=	6
	α err prob	=	0.2322430
	β err prob	=	0.2322430
	**Power (1−β err prob)**	**=**	**0.7677570**

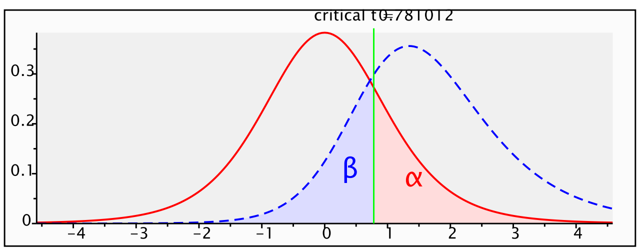

Distribution Plot of Oocyst Onset

Supplementary Material 2

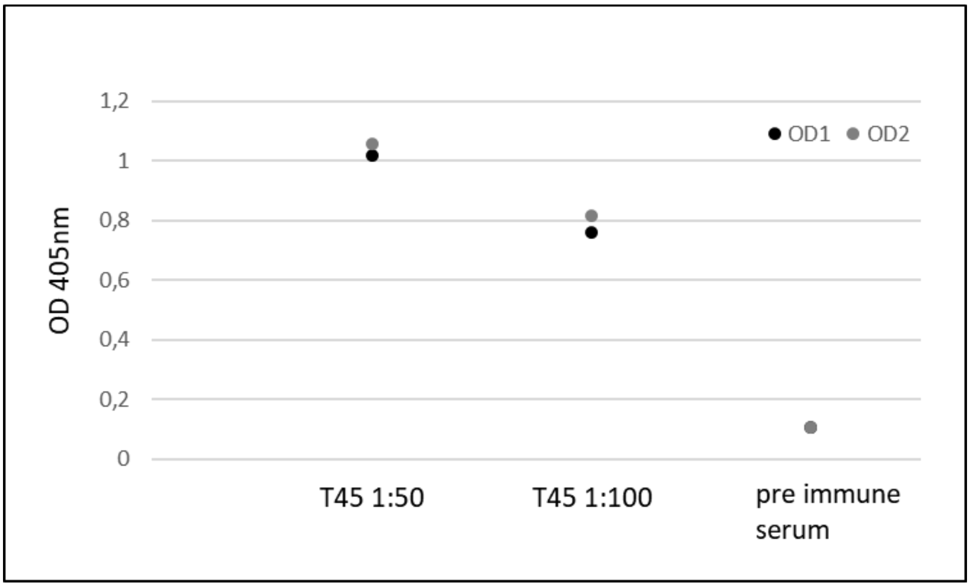

Figure S1. IgY Ab response in serum of laying hen immunized with *C. parvum* APCH-P23 antigen. This ELISA was performed using oocyst from fecal samples to confirm the specificity of the IgY antibody to p23 in the oocyst context. T45: timepoint 45 after the first immunization. OD1 and OD2 are replicates of the same serum sample.
